# MicroRNA-143-5p targeting eEF2 gene mediates intervertebral disc degeneration through the AMPK signaling pathway

**DOI:** 10.1186/s13075-019-1863-5

**Published:** 2019-04-15

**Authors:** Qi Yang, Xiao-Peng Guo, Yan-Li Cheng, Yang Wang

**Affiliations:** 1 The 3rd Ward of Department of Orthopedics Surgery, Taihe Hospital, Shiyan, 442000 People’s Republic of China; 2 Department of Dermatology, Taihe Hospital, Shiyan, 442000 People’s Republic of China; 3 The 2nd Ward of Department of Neurosurgery, Taihe Hospital, No. 32, Renmin South Road, Maojian District, Shiyan, 442000 Hubei Province People’s Republic of China

**Keywords:** MicroRNA-143-5p, EEF2, AMPK signaling pathway, Nucleus pulposus cells, Differentiation, Apoptosis, Senescence

## Abstract

**Background:**

Intervertebral disc degeneration (IDD) is a major contributor to back, neck, and radicular pain, and the treatment of IDD is costly and relatively ineffective. Dysregulation of microRNAs (miRNAs) has been reported to be involved in IDD. The purpose of our study is to illustrate the potential that miR-143-5p targeting eEF2 gene mediates IDD.

**Methods:**

Following the establishment of the IDD rat models, expression of miR-143-5p, eEF2, Bcl-2, Bax, AMPK, mTOR, cyclinD, COL2, ACAN, and DCN was detected. The NP cells isolated from degenerative intervertebral disc (IVD) were introduced with a series of mimic, inhibitor, or AICAR to explore the functional role of miR-143-5p in IDD and to characterize the relationship between miR-143-5p and eEF2. Cell viability, cell cycle, apoptosis, and senescence were also evaluated.

**Results:**

A reduction in eEF2, an increase in miR-143-5p, and activation of the AMPK signaling pathway were observed in degenerative IVD. Moreover, increased senescent NP cells were observed in degenerative IVD. eEF2 was confirmed as a target gene of miR-143-5p. miR-143-5p was found to activate the AMPK signaling pathway. The restoration of miR-143-5p or the activation of AMPK signaling pathway decreased COL2, ACAN, and DCN expression, coupled with the inhibition of NP cell proliferation and differentiation, and promotion of NP apoptosis and senescence. On the contrary, the inhibition of miR-143-5p led to the reversed results.

**Conclusion:**

The results demonstrated that the inhibition of miR-143-5p may act as a suppressor for the progression of IDD.

## Background

Intervertebral disc degeneration (IDD) is a common complex disease of the spine, leading to musculoskeletal disability and poor quality of life for patients [1, 2]. IDD is often accompanied with low back pain (LBP), radiculopathy, or myelopathy, and its lifetime incidence rate is greater than 90% [3]. Prominent changes occur during IDD, including loss of extracellular matrix, altered phenotype of normal disc cells, and the release of pro-inflammatory cytokines [4]. It is interesting to note that nucleus pulposus (NP) was responsible for maintenance of disc function and structure [5]. The accelerated apoptosis and senescence of NP cells were found to be a possible cause for IDD [6]. Protection against NP cell apoptosis and senescence may be conducive for the amelioration of IDD [7, 8]. A previously conducted study demonstrated that reinsertion of activated NP cells can delay the process of disc degeneration [9]. This prompted us to improve understanding of the biology of the intervertebral disc (IVD) healing and to identify strategies to enhance the regenerative process. In previous investigations, microRNAs (miRNAs) were reported for their deregulation in NP cells, with effect on the proliferation and apoptosis of NP cells, or their relationship with breakdown of balance between (ECM) synthesis and degradation in NP cells in IDD [10–12].

MiRNAs can downregulate the gene expression by targeting mRNAs for translational repression and/or cleavage, which enables them critical roles in cellular processes such as proliferation, invasion, apoptosis, and senescence [13]. A previous study has confirmed that miR-143-5p plays a significant role in epithelial-mesenchymal transition (EMT) and metastasis of gallbladder cancer [14]. Besides, it has also been proven to involve in the progression and prognosis of cervical cancer [15]. Furthermore, recent evidence has revealed that miR-143 is upregulated in degenerative disc tissues [16]. A study conducted by Mu et al. found that miR-143 affects proliferation and apoptosis of human hypertrophic scar fibroblasts (HSFs) and inhibits ECM production-associated protein through suppression of the Akt/mTOR pathway [17]. As was previously reported, eukaryotic elongation factor 2 (eEF2) is a target of mTOR [18], and thus it was speculated that miR-143 could modulate eEF2. Furthermore, eEF2 has been predicted to be a target of miR-143-5p by using the online prediction program. The eEF2 gene has been proven to be a common calcium/calmodulin (Ca/CaM)-dependent Ser/Thr-kinase that is activated by mitotic agents involved in cell proliferation and apoptosis [19]. eEF2 kinase (eEF2K), an enzyme that inactivates eEF2, is activated by AMP-kinase (AMPK) and contributes to cell survival [20, 21]. Tumor cells exploit this pathway to adapt to nutrient deprivation via reactivating the AMPK-eEF2K signaling pathway [22]. Recently, Wang et al. have provided evidence showing that resveratrol has the ability to promote NP cell autophagy via activation of AMPK signaling pathway, thus acting as a novel preventive role in IDD [23]. Based on the aforementioned literature, a hypothesis can be drawn that miR-143-5p is involved in IDD via the AMPK signaling pathway by regulating eEF2. In this present study, the functions of miR-143-5p on NP cells of IDD rats were explored, as was the underlying mechanism involving the AMPK signaling pathway.

## Methods

### Ethics statement

This study was carried out in strict accordance with the recommendations in the Guide for the Care and Use of Laboratory Animals. All efforts were made to minimize suffering of the animals. The experimental procedures were approved by the Animal Ethics Committee of Taihe Hospital.

### Study subjects

Fifteen male Lewis rats, weighing 297 to 323 g and aged 12 to 14 weeks, were purchased from the Laboratory Animal Research Center of Southern Medical University (Guangzhou, China). A week before treatment, the rats were housed with free access to water and food, under a 12-h light/dark cycle with the environment at 22~24 °C with the humidity of 50~60%.

### Establishment of IDD model

IDD models were developed as previously reported [24]. In brief, 15 male Lewis rats aged 12~14 weeks were selected for the experiments. Rat coccygeal vertebrae (Co6/Co7 and Co7/Co8) were punctured using the 18-G needle, in order to establish the model of IDD. Besides, the rats (Co8/Co9) without puncture were regarded as the control group. The central NP (nucleus pulposus), 5 mm distant from the skin, was the object of puncture. The pinpoint was rotated for 360° and kept for 30 s. Images of X-ray and magnetic resonance imaging (MRI) were obtained of every rat coccygeal vertebra before the puncture and in the second week and the fourth week after the puncture. The disc height index (DHI) (%) and rate of disc height change (RDHC) were calculated [24]. The degeneration degree of caudal IVD of rats was graded as follows: I was normal, II was slight IDD, III was moderate, and IV was serious [25], and the rate of IDD was calculated as previously described [26]. All IDD grades and imaging analysis were conducted by three independent surgeons, twice respectively so as to evaluate the reliability of the rating system. The rats with typical IDD, which was confirmed through imaging, were all euthanatized by CO_2_ inhalation 4 weeks later. IDD specimen, obtained after dissecting the rats, was used for hematoxylin-eosin (HE) staining and immunohistochemistry.

### HE staining

Parts of degenerative IVD tissues (Co6/Co7 or Co7/Co8) and normal IVD tissues (Co8/Co9) were extracted for the HE staining. The target IVD tissues (Co7-Co8 and Co8-Co9) and adjacent caudal vertebrae were excised, then fixed with 3% neutral formaldehyde, embedded with paraffin, and cut into 5-μm-thick sections. Following the cutting period, the tissue sections were dewaxed with xylene twice (each time for 5 min), then rehydrated with 100%, 95%, 80%, and 75% gradient ethanol respectively for 1 min and rinsed for 2 min under running water. Sections were then stained with hematoxylin for 2 min and rinsed with running water for 10 s, followed by color separation with 1% hydrochloric acid ethanol for 10 s. Then sections were then rinsed by distilled water for 1 min and stained with eosin for 1 min. Following the eosin staining periods, sections were dehydrated by 95% and 100% ethanol for two times (each time 1 min) after being washed by distilled water for 10 s. Finally, sections were permeabilized by xylene and then mounted by neutral balsam.

### Safranin O staining

The tissues were fixed with 4% paraformaldehyde, dehydrated, permeabilized, and then paraffin embedded. The paraffin-embedded tissues were sliced into 5-μm-thick sections. After this, the tissue sections were dewaxed and stained conventionally using hematoxylin and eosin. Following that, the sections were stained with 0.5% Safranin O for 5 min. After washing under distilled water, the tissue sections were dehydrated twice using 95% and 100% gradient ethanol (2 min each time). After that, the sections were permeabilized using xylene and sealed with neutral balsam.

### Immunohistochemistry

Degenerative IVD and normal IVD tissues were respectively fixed with 10% formalin, embedded into paraffin, and cut into 4-μm sections. The tissue sections were then baked at 60 °C for 20 min; dewaxed with xylene I, xylene II, and xylene III (10 min for each); immersed in absolute ethanol I, absolute ethanol II, and 90% water-ethanol (2 min for each); treated with 3% H_2_O_2_ for 15 min; and then washed by distilled water three times (each time for 2 min). The tissue sections then underwent microwave antigen retrieval. Following the antigen revival period, the sections were immersed in the 0.01 M citrate buffer solution (pH 6.0) and heated in a microwave oven until boiling. After 5 min, sections were further heated until boiling. After another 5-min period, sections were cooled down to room temperature. Then, the sections were blocked with 10% bovine serum albumin at 37 °C for 30 min. After the surplus serum was discarded, primary antibody to eEF2 (1:2000, ab75748, Abcam, Cambridge, MA, USA) was added into sections until they were completely covered, and incubated at 4 °C overnight. After that, sections were incubated at 37 °C for 1 h and then washed by 0.02 M phosphate buffered saline (PBS) three times (each time for 5 min). After being washed via PBS, sections were added with secondary antibody and goat anti-rabbit immunoglobulin G (IgG) (DF7852, Shanghai Yao Yun Biotechnology Co., Ltd., Shanghai, China) for incubation at 37 °C for 30 min, and washed with 0.02 M PBS three times (each time for 5 min). Sections were added with a horseradish peroxidase-labeled streptomycin avidin reagent, incubated at 37 °C for 30 min, and then washed with 0.02 PBS three times (each time for 5 min). Following that, sections were developed by diaminobenzidine (DAB) under conditions void of light, which was controlled under the microscope for 5 min, and then fully washed by distilled water. After being washed by distilled water, sections were counterstained with hematoxylin for 1 min, conventionally dehydrated by ethanol and permeabilized, and sealed with neutral gum. The staining was observed under a microscope, with the brownish yellow particles regarded as positive. Five high-power visual fields (× 400) were randomly chosen from each section with 100 cells counted in each field. The mean values of percentage of positive cells in total cells were then calculated. The experiment was performed three times.

### Cell culture

Following a successful model establishment for 4 weeks, 4 rats were given lethal doses of CO_2_, with the spine separated to expose normal and IDD spine under an aseptic condition. The fibrous ring was cut with a scalpel to separate gelatinous nucleus, which then was rinsed in sterile D-hanks three times to remove bloodstain. NP tissues were cut into blocks at a size of 1 × 1 × 1 mm and placed into a centrifuge tube. After that, the tube was added with twofold volume of 0.1% type II collagenase, placed in a water bath at 37 °C for digestion, and then shaken every 10 min. After the digestion period, the tissues were centrifuged at 179×*g* for 5 min with the supernatant removed, and detached with 10 U/mL hyaluronidase in a water bath for 2 h. Tissues were centrifuged at 179×*g* for 5 min and then washed by Dulbecco’s modified Eagle’s medium (DMEM)-F12 three times. Following the counting period, cells were inoculated into 25-cm culture flasks at 1 × 10^6^, added with DMEM-F12 containing 100 U/mL streptomycin and 15% fetal bovine serum (FBS), and cultured in a cell incubator with 5% CO_2_ at 37 °C. The medium was changed after a week and then changed every 3 days. Following a 20-day period, when the cells were detached from the wall, a fibroblast region with long spindle and polygon-shaped cells was scrapped with cell scraper and then suspended in culture medium. Meanwhile, the circular and short shuttle-shaped NP cells were retained under a phase-contrast microscope. Afterwards, the flask was rinsed with culture medium two times and continually cultured. Until the circular and short shuttle-shaped NP cells turned into the clone population with single morphology, the cells were treated with 0.25% trypsin for resuspension, and then inoculated into another culture flask for further culture.

### Cell transfection and grouping

The abovementioned cultured cells were grouped into control group (NP cells from normal IVD), blank group (NP cells from degenerative IVD without any transfection), negative control (NC) group (NP cells from degenerative IVD transfected with miR-143-5p negative control sequence), miR-143-5p mimic group (NP cells from degenerative IVD transfected with miR-143-5p mimic), miR-143-5p inhibitor group (NP cells from degenerative IVD transfected with miR-143-5p inhibitor), AICAR group (NP cells from degenerative IVD added with 0.5 mmol/L AICAR, AMPK signaling pathway activator), and miR-143-5p inhibitor + AICAR group (NP cells of degenerative IVD added with 0.5 mmol/L AICAR and transfected with miR-143-5p inhibitor). Twenty-four hours before the transfection, cells were inoculated into a six-well plate. The transfection was conducted based on the instructions of lipofectamine 2000 (11668-019, Invitrogen, Carlsbad, CA, USA) when the cell confluence reached 30 to 50%. The 100 pmol aliquots of miR-143-5p mimic, miR-143-5p inhibitor, miR-143-5p inhibitor + AICAR, and AICAR were all separately diluted with 250 μL of serum-free Opti-MEM (51985042, Gibco, Gaitherburg, MD, USA) to the final concentration of 50 nM, followed by incubation at room temperature for 20 min. A 5-μL aliquot of lipofectamine 2000 was diluted with 250 μL serum-free medium, followed by incubation at room temperature for 5 min. The abovementioned two mixtures were mixed completely and allowed to incubate at room temperature for 20 min. Following this, the cells were incubated with the mixture in an incubator with 5% CO_2_ at 37 °C for 6 to 8 h. Then, the medium was changed into the complete medium in order to culture for another 24 to 48 h. The pertinent sequences are shown in Table 1.

**Table 1:** Mimic or inhibitor sequence for cell transfection

Plasmid	Sequence(5′–3′)
miR-143-5p mimic	5′-CCACGTCACGACGTAGAGACC-3′
miR-143-5p inhibitor	5′-CCAGAGATGCAGCACTGCACC-3′
miR-143-5p NC	5′-UCCUCCGAACGUGUCACGUTT-3′

Note: miR-143-5p, microRNA-143-5p

*NC* negative control

### Dual luciferase reporter gene assay

The target genes of miR-143-5p were analyzed by biology prediction website Targetscan (http://www.targetscan.org). HEK-293T cells (AT-1592, ATCC, Manassas, VA, USA) were plated into a 24-well plate and cultured for 24 h. The total RNA of cells was extracted and reversely transcribed into cDNA. The full-length sequence of eEF2 3′-UTR was obtained by polymerase chain reaction (PCR) amplification with cDNA as the template. According to the sequence of eEF2, the primers were designed (forward primer: 5′-ATGAGGGCAAGATGAAGCTG-3′, and reverse primer: 5′-ATGAAGGACGGGATGTTCAC-3′) and amplified with genome extracted from HEK-293T cells as a template. Following the amplification period, the primers were digested by enzyme and cloned into the downstream of pmiRRB carrier luciferase coding gene to obtain eEF2 dual luciferase reporter (DLR) vector, namely pmiRRB-eEF2-3′UTR, which was separately co-transfected with miR-143-5p vector, siRNA-miR-143-5p, or negative control into HEK-293T cells. After transfection for 48 h, the culture medium was aspirated, and cells were rinsed twice with PBS. Then cells were then collected and lysed. The luciferase activity was measured with Dual-Luciferase® Reporter Assay System (E1910, Promega, Madison, WI, USA). A total of 50 μL of firefly luciferase working fluid was added into every 10-μL aliquot of cells to examine firefly luciferase activity. Then a 50-μL aliquot of renilla luciferase working liquid was added to examine renilla luciferase activity. The ratio of firefly luciferase activity to renilla luciferase activity represented the relative luciferase activity. The experiment was repeated three times.

### Reverse transcription quantitative polymerase chain reaction (RT-qPCR)

The total RNA was extracted from the tissues using the Trizol extraction kit (15596-018, Invitrogen, Grand Island, NY, USA), and the total RNA was extracted from cells using miRNeasy total RNA extraction kit (217004, Beijing Huaxia Ocean Technology Co., Ltd., Beijing, China). The ratio of A260/A280 and concentration of RNA were determined using a Nanodrop ultraviolet spectrophotometer (2000, Thermo, Waltham, MA, USA), with RNA stored at − 80 °C. Following that, the total RNA was reversely transcribed into cDNA according to the instructions of Applied Biosystems StepOneTM and StepOnePlusTM Real-Time PCR Systems (4379704, Applied Biosystems, Foster City, CA, USA). The RT-qPCR assay kit was purchased from Ambion Corporation (NY, CA, USA). RT-qPCR was conducted with PCR instrument (AM1005, Invitrogen, NY, CA, USA), and the conditions were as follows: pre-denaturation at 95 °C for 3 min and 35 cycles of denaturation at 95 °C for 15 s, annealing at 60 °C for 30 s, and extension at 72 °C for 30 s. The glyceraldehyde-3-phosphate dehydrogenase (GAPDH) was used as the internal reference of eEF2, mammalian target of rapamycin (mTOR), AMP activated protein kinase (AMPK), B cell lymphoma-2 (Bcl-2), and cyclinD. Besides, U6 was used as the internal reference of miR-143-5p. The primers (Table 2) were synthetized by Shanghai Boya Bio Technology Service Co. Ltd. (Shanghai, China). Every experiment was repeated three times. The reliability of PCR results was evaluated by solubility curve, and the cycle threshold (CT) value (inflection of amplification dynamic curve) was obtained. The relative expression of target gene was calculated by a 2^−ΔΔCT^ method [27]. The formula was as follows: ΔΔCt = [Ct (target gene) − Ct (reference gene)] _experimental group_ − [Ct (target gene) − Ct (reference gene)] _control group_. This method was applicable for cell experiments.

**Table 2:** Primer sequence for RT-qPCR

Gene	Sequence
miR-143-5p	F: 5′-GCATCTCTGGTCAGTTGGG-3′
	R: 5′-GACCTCAAGAACAGTAT-3′
eEF2	F: 5′-GAGCTCTCCGAGAACGACC-3′
	R: 5′-TACAGTGCCCAGGACAGGAT-3′
mTOR	F: 5′-TTGAGGTTGCTATGACCAGAGAGAA-3′
	R: 5′-TTACCAGAAAGGACACCAGCCAATG-3′
AMPK	F: 5′-ATCCGCAGAGAGATCCAGAA-3′
	R: 5′-CGTCGACTCTCCTTTTCGTC-3′
Bcl-2	F: 5′-CCTGCCCCAAACAAATATGAAAAG-3′
	R: 5′-TTGACCATTTGCCTGAATGTGTG-3′
cyclinD	F: 5′-TCCGCAAGCATGCACAGA-3′
	R: 5′-GGTGGGTTGGAAATGAACTTCA-3′
Bax	F: 5′-CGTCTGGCCCTGTATGTCTA-3′
	R: 5′-CAACCACCTCTCTGTGCAAT-3′
U6	F: 5′-CTCGCTTCGGCAGCACA-3′
	R: 5′-AACGCTTCACGAATTTGCGT-3′
GAPDH	F: 5′-CGTGATCGAGGGCTGTTGG-3′
	R: 5′-CTGCTTCAGTTGGCCTTTCG-3′

Note: *RT-qPCR* reverse transcription quantitative polymerase chain reaction, *miR-143-5p* microRNA-143-5p, *eEF2* eukaryotic translation elongation factor 2, *AMPK* AMP activated protein kinase, *Bcl-2* B cell lymphoma-2, *mTOR* mammalian target of rapamycin, *GAPDH* glyceraldehyde-3-phosphate dehydrogenase, *F* forward, *R* reverse, *Bax* Bcl-2 Associated X protein

### Western blot analysis

Total protein was extracted using a total protein extract kit (R0010, Beijing Solarbio Technology Co. Ltd., Beijing, China). The transfected cells were washed using the precooling PBS three times. The protein lysate (60% radio immunoprecipitation assay [RIPA] lysis buffer + 39% sodium dodecyl sulfate [SDS] + 1% protease inhibitors) was added into every cell bottle, and the mixture was lysed on ice for 30 min in the EP tube. Then cells were centrifuged at 32,608×*g* in the high-speed freezing centrifuge for 30 min at 4 °C, and the supernatant was collected and placed on the icebox to measure protein concentration using bicinchoninic acid (BCA) method. Subsequently, 10% separation gel and 5% spacer gel were prepared using sodium dodecyl sulfate-polyacrylamide gel electrophoresis (SDS-PAGE) kit, and the protein was separated using electrophoresis on polyacrylamide gel. Following that, protein was transferred onto a nitrocellulose membrane. The membrane was blocked with 5% bovine serum albumin (BSA) at room temperature for 1 h and incubated with primary antibodies of rabbit anti eEF2 (1:10000, ab75748), p-AMPK (1:2000, ab133448), Bax (1:1000, ab32503), p-mTOR (1:5000, ab137133), cyclinD (1:10000, ab134175), Bcl-2 (1:1000, ab196495), t-AMPK (1:2000, ab32047), t-mTOR (1:2000, ab2732), and GAPDH (1:1000, ab9485). All the above antibodies were purchased from Abcam Inc. (Cambridge, MA, USA). The following day, the membrane was incubated with the secondary antibody, goat anti-rabbit IgG (1:2000, ab205718, Abcam Inc., Cambridge, MA, USA) at 4 °C for 1 h. The proteins were visualized with a developing agent and imaged using a Bio-rad imaging system (MG8600, Beijing Thmorgan Biotechnology Co., Ltd., Beijing, China). Quantitative analysis was conducted using IPP7.0 software (Media Cybernetics, Silver Springs, MD, USA). The ratio of gray value of eEF2, p-AMPK, p-mTOR, Bcl-2, cyclinD, Bax, t-AMPK, and t-mTOR protein bands to that of the internal reference GAPDH protein band represented the protein levels of genes respectively. The procedures were also applicable for cell experiments.

### 3-(4,5-Dimethylthiazol-2-yl)-2,5-diphenyltetrazolium bromide (MTT) assay

The transfected cells in each group were incubated in RPMI 1640 culture medium containing 10% FBS based on the predetermined concentration with 5% CO_2_ at 37 °C for 48 h. Cells in the logarithmic growth phase were collected for the following experiment. The cell suspension was transferred into a centrifuge tube and then triturated evenly using a sterile straw. Single-cell suspension was stained by trypan blue staining solution. The number of living cells was counted. The living cells were seeded into a 96-well plate (180 μL of cells per well) at a density of 1 × 10^4^ cells/well. Afterwards, cells were cultured at 37 °C with 5% CO_2_ for 16 to 48 h. Each well was incubated with 20 μL of 5% MTT under conditions void of light. The 96-well plate was cultured in a 5% CO_2_ incubator at 37 °C under conditions void of light for 4 h. After that, the cells were centrifuged at 179×*g* for 10 min with the supernatant removed. Then, 100 μL dimethyl sulfoxide (DMSO) solution was used to dissolve the crystal fully under conditions void of light for 10 min. Fifteen minutes later, optical density (OD) value was detected at 570 nm using enzyme-linked immunosorbent assay (ELISA) reader (SAF-680T, Multiskan GO, Thermo, Waltham, MA, USA). Cell growth curve was plotted with time as the abscissa and OD value as the ordinate.

### Flow cytometry

After transfection of 24 h, the cells were collected, washed with cold PBS three times, centrifuged with the supernatant removed, and then resuspended with PBS to adjust cell density to 1 × 10^5^/mL. Cells were then fixed by 1 mL of 75% ice ethanol (precooled at − 20 °C) at 4 °C for 1 h. After that, they were centrifuged and washed with PBS two times. Cells were incubated with 100 μL RNase A under conditions void of light, water-bathed at 37 °C for 30 min, and stained with 400 μL of propidium iodide (PI) (D0820, Sigma, San Francisco, CA, USA). They were incubated at 4 °C under conditions void of light for 30 min. After that, the red fluorescence at the wavelength of 488 nm was recorded by flow cytometry (Gallios, Beckman Coulter, S.Kraemer Boulevard Brea, CA, USA) to detect cell cycle. After transfection for 48 h, cells were treated with ethylenediamine tetraacetic acid (EDTA)-free trypsin and centrifuged to discard the supernatant. Cells were rinsed by cold PBS three times and centrifuged with the supernatant discarded. In accordance with the instructions of Annexin-V-FITC kit (4030ES20, Sigma, SF, CA, USA), the Annexin-V-FITC/PI staining solution was prepared with Annexin-V-FITC, PI, (2-hydroxyethyl)-1-piperazineethanesulfonic acid (HEPES) in the proportion of 1:2:50. The 1 × 10^6^ cells were resuspended in every 100-μL aliquot of staining solution. Cells were then incubated at room temperature for 15 min and then added with 1 mL of HEPES. The cells were excited at 488 nm, the FITC fluorescence was measured at 525 nm, and PI fluorescence was measured at 620 nm with a band-pass filter.

### Senescence-associated β-galactosidase (SA-β-gal) staining

The senescence of NP cells was assessed by SA-β-gal staining. NP cells in the control group and experimental groups were collected and seeded into six-well plates at a density of 3 × 10^5^ cells/well. Upon reaching 70% confluence, cells were washed using PBS and fixed by 1 mL SA-β-gal staining solution for 15 min at room temperature. The cells were washed three times by PBS (3 min per wash). The cells were stained with 1 mL SA-β-gal staining solution and then sealed with a preservative film at 37 °C overnight. The SA-β-gal-stained cells (the cytoplasm stained in dark blue) and total cells were counted under an optical microscope. The SA-β-gal-positive rate of NP cells = the number of SA-β-gal-stained NP cells/the number of total cells. The cytoplasm of SA-β-gal-positive cells presented dark blue staining.

### Statistical analysis

Statistical analysis was conducted using SPSS 17 (SPSS, Chicago, IL, USA), and data were expressed by mean ± standard deviation. Comparison of data between two groups was conducted using a *t-*test and that of data among multiple groups was performed by one-way analysis of variance (ANOVA). Disc height index and rate of disc height change were analyzed by one-way ANOVA. The grade of IDD was analyzed by kappa. If the kappa value was 0–0.2, it means the consistency deviation was comparatively large and the result was very poor. If the kappa value was 0.21–04, it means the result was poor. If the kappa value was 0.41–0.60, it means the result was ordinary. If the kappa value was 0.61–0.8, it means the consistency of the result was comparatively good. Besides, if the kappa value was over 0.81, the result was basically the same and the result was reliable. *p* < 0.05 was considered statistically significant.

## Results

### IDD models are successfully established

Among the 30 rat coccygeal vertebras with puncture, IVD of 16 rats degenerated in the second week after puncture at a rate (modeling success rate) of 53%. In the fourth week, the degenerated number increased to 25 with a rate of 83%. In the second week after puncture, the mean DHI (%) was 86.44 ± 3.00, RDHC was 0.75, and the mean score of IDD was 3.35, with an obvious difference compared with that before the puncture. In the fourth week after puncture, the mean DHI (%) was 69.00 ± 2.37 and the RDHC was 0.7 which was significantly lower when compared to that of the second week. T2-weighted imaging demonstrated that the IVD construction was damaged and that the signal was weak with the color of bright white turning into gloomy gray along with the diminished or collapsed disc height. Also, in the fourth week, the mean score of the IDD was 5.07, and the degree of degeneration became worse after the puncture. According to the data shown in Table 3, in the second week after puncture, the mean DHI (%) was 86.44 ± 3.00 was significantly reduced when compared with that before puncture. The mean RDHC was 0.75 ± 0.03 with statistical difference compared with the value before puncture. In the fourth week after puncture, the mean DHI (%) was 69.00 ± 2.37 (*p* < 0.001) and the mean RDHC was 0.70 ± 0.02 (*p* < 0.001), which statistically differed from former data before the puncture.

**Table 3:** Degeneration of rat caudal IVD at different time points

Time point	Non-degenerative IVD	Total degenerative IVD	Degenerative score	Degenerative rate	DHI (%)	RDHC
At the beginning	30	0	0	0	100 ± 0	1
The 2nd week	14	16	3.35 ± 0.30^*^	53%^*^	86.44 ± 3.00^*^	0.75 ± 0.03^*^
The 4th week	5	25	5.07 ± 0.41^*^	83%^*^	69.00 ± 2.37^*^	0.70 ± 0.02^*^

Note: *n* = 15

*DHI* disc height index, *RDHC* rate of disc height change, *IVD* intervertebral disc

^*^p < 0.05 vs. the data obtained at the beginning; the comparison of data was conducted using a *t-*test

**Fig. 1:**
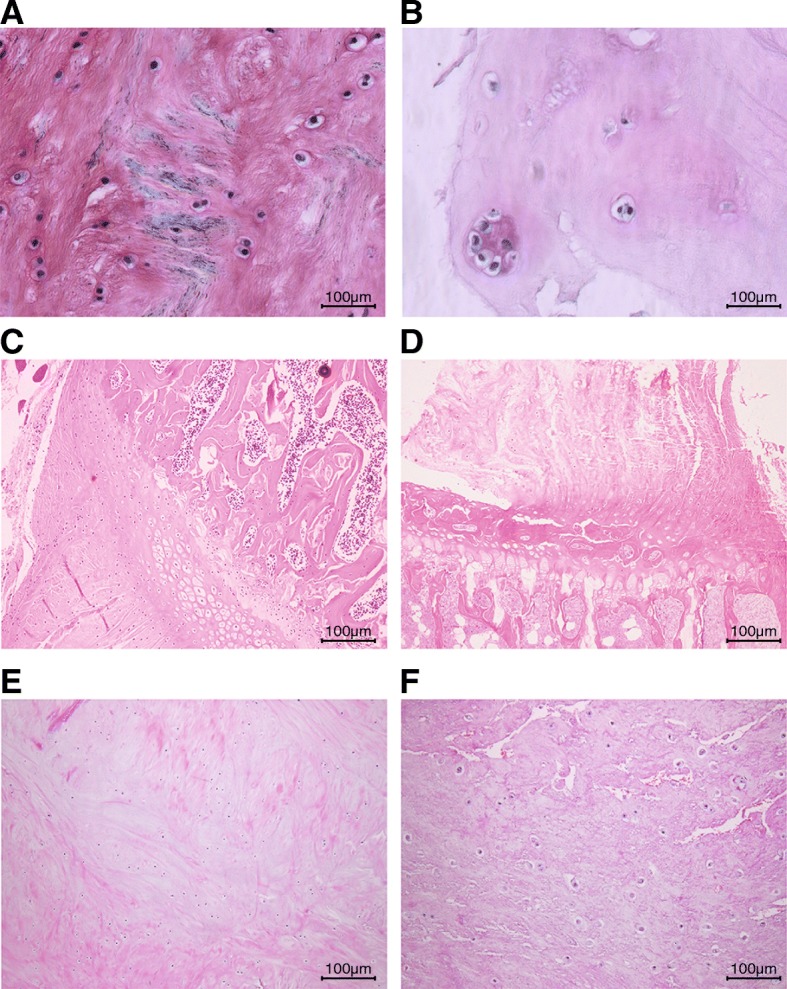
The induction of IDD in rats exhibits degenerative IVD and shrank NP. **a** Safranin O staining images of normal IVD tissues and NP (× 100). **b** Safranin O staining images of degenerative IVD tissues and NP. **c** HE staining images of normal IVD tissues. **d** HE staining images of degenerative IVD tissues. **e** HE staining images of NP in normal IVD tissues. **f** HE staining images of NP in degenerative IVD tissues. HE, hematoxylin-eosin; IVD, intervertebral disc; IDD, intervertebral disc degeneration; NP, nucleus pulposus

Morphological characteristics of IVD tissues were observed after Safranin O staining and HE staining. As depicted in Fig. 1a, b, the cartilage of normal IVD tissues exhibited a clear and regular structure, with a large area of red staining in the NP and cartilage endplate, and rich proteoglycans. By contrast, degenerative IVD tissues showed a disordered structure, lighter red staining, narrowed IVD space, and derangement of annulus fibrosus. HE staining results in Fig. 1c–f displayed that in rat caudal IVD, the nearly round complete NP occupied more than half area of the IVD, and the stellate NP cells were equally distributed in the nucleus pulposus. The IVD degenerated in the IDD rats with NP shrank, and the NP cells exhibited vacuolar degeneration with cluster distribution. Taken together, IDD had a close correlation with the IVD degeneration and NP shrank.

### eEF2 protein is lowly expressed in degenerative IVD tissues

**Fig. 2:**
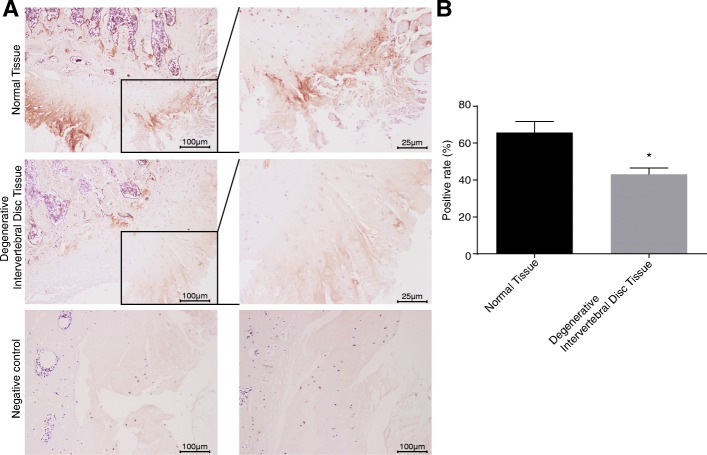
eEF2 protein is expressed at a low level in degenerative IVD. **a** Immunohistochemistry images of eEF2 protein expression in normal IVD and degenerative IVD (× 100 in left panel, × 400 in right panel). eEF2-positive particle is stained brownish yellow in notochord cells and chondrocytes. **b** Positive rate of eEF2 protein expression in normal IVD and degenerative IVD; *n* = 15; positive rate represents the mean value of the percentage of positively stained cells in total cells; data were expressed by mean ± standard deviation; comparison of data between two groups was conducted using a *t-*test; *, *p* < 0.05 vs. normal IVD; IVD, intervertebral disc; IDD, intervertebral disc degeneration; eEF2, eukaryotic elongation factors-2

Following the establishment of the IDD models, immunohistochemistry was used for examining the eEF2 protein positive rate, so as to shed light on the effect of eEF2 in IDD. The result revealed that a eEF2-positive particle was brownish yellow in notochord cells and chondrocytes. The positive rate of eEF2 protein expression in NP cells of the normal IVD was 65.7 ± 6.0%, while being 43.1 ± 3.5% in the degenerative IVD. The positive rate of eEF2 protein expression in the normal IVD was much higher than that in degenerative IVD *(p* < 0.05) (Fig. 2a, b). Based on the result, it was concluded that eEF2 protein expressed at a low level in degenerative IVD.

### IDD is associated with increased NP cell senescence

**Fig. 3:**
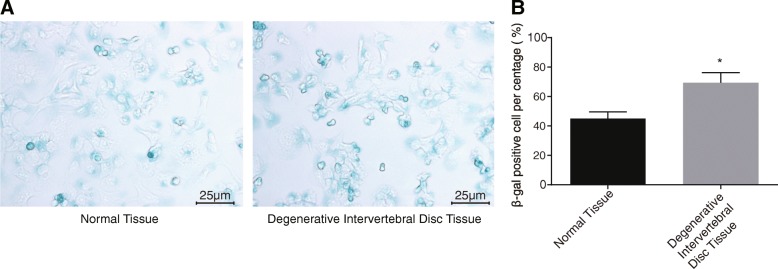
Degenerative IVD shares a close association with increased NP cell senescence. **a** SA-β-gal staining images of senescent NP cells in normal IVD and degenerative IVD under a microscope (× 400); dark blue-stained cells were senescent ones. **b** The positive rate of SA-β-gal staining in normal IVD and degenerative IVD; the positive rate of SA-β-gal staining represents the percentage of dark blue-stained NP cells in total NP cells; the experiment was repeated three times independently; data were expressed by mean ± standard deviation; comparison of data between two groups was conducted using a *t-*test; *, *p* < 0.05 vs. normal IVD; IVD, intervertebral disc; NP, nucleus pulposus; SA-β-gal, senescence-associated β-galactosidase

SA-β-gal staining was carried out to observe senescent changes in NP cells. As shown in Fig. 3, the cytoplasm in normal IVD was stained dark blue without clear vacuoles and no obvious staining in the nucleus. The degenerative IVD tissues showed a much higher positive staining rate than the normal IVD tissues (*p* < 0.01). Therefore, it was concluded that IVD degeneration had a close correlation with increased NP cell senescence.

### miR-143-5p and AMPK are upregulated while eEF2 is downregulated and cartilage differentiation is inhibited in degenerative IVD tissues

**Fig. 4:**
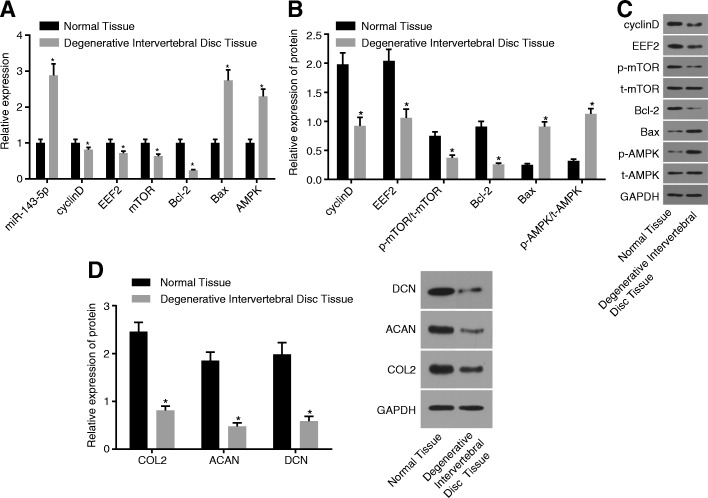
IDD rats exhibit upregulated miR-143-5p and activated AMPK signaling pathway yet inhibited NP cell differentiation and eEF2. **a** miR-143-5p expression and mRNA expression of Bax, AMPK, mTOR, Bcl-2, cyclinD, and eEF2 in normal IVD and degenerative IVD. **b**, **c** Protein levels and bands of Bax, AMPK, mTOR, Bcl-2, cyclinD, and eEF2 in normal IVD and degenerative IVD. **d** Protein levels of COL2, ACAN, and DCN in normal IVD and degenerative IVD; the experiment was repeated three times independently; data were expressed by mean ± standard deviation; comparison of data between two groups was conducted using a *t-*test; ^*^, *p* < 0.05 vs. normal IVD; miR-143-5p, microRNA-143-5p; IVD, intervertebral disc; eEF2, eukaryotic translation elongation factor 2; AMPK, AMP activated protein kinase; Bax, Bcl-2 Associated X protein; Bcl-2, B cell lymphoma-2; mTOR, mammalian target of rapamycin; GAPDH, glyceraldehyde-3-phosphate dehydrogenase; COL2, collagen type II; ACAN, aggrecan; DCN, decorin

The mRNA and protein levels of cartilage differentiation-related and AMPK signaling pathway-related genes in normal IVD and degenerative IVD were detected using RT-qPCR (Fig. 4a) and Western blot analysis (Fig. 4b, c). When compared to the normal IVD, degenerative IVD showed significantly decreased mRNA and protein levels of mTOR, Bcl-2, cyclinD and eEF2, and p-mTOR/t-mTOR protein level ratio, while obviously increased mRNA and protein levels of Bax and AMPK, p-AMPK/t-AMPK protein level ratio, and miR-143-5p expression.

The protein levels of cartilage differentiation-related genes in normal IVD and degenerative IVD were detected using Western blot analysis (Fig. 4d). When compared to the normal IVD, degenerative IVD showed significantly decreased levels of COL2, ACAN, and DCN proteins (*p* < 0.05).

Therefore, these results demonstrated that miR-143-5p highly expressed and AMPK signaling pathway activation was induced in degenerative IVD, while eEF2 lowly expressed and cartilage differentiation was inhibited.

### eEF2 is a target gene of miR-143-5p

**Fig. 5:**
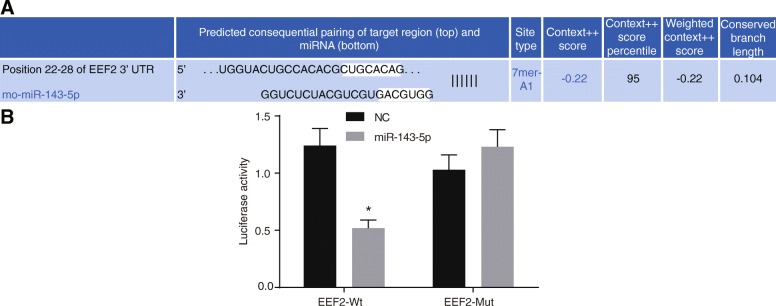
miR-143-5p directly targets eEF2. **a** Putative binding site of miR-143-5p to the 3′UTR of eEF2 predicted using Targetscan. **b** The luciferase activity detection using vectors constructed with eEF2-3′UTR in the presence of miR-143-5p mimic or NC; three replicates were settled in each group and the experiment was repeated three times independently; data were expressed by mean ± standard deviation; comparison of data between two groups was conducted using a *t-*test; ^*^, *p* < 0.05 vs. the NC group; miR-143-5p, microRNA-143-5p; eEF2, eukaryotic elongation factors-2; 3′UTR, 3′untranslated regions; Wt, wild-type; Mut, mutant; NC, negative control; ANOVA, analysis of variance

Bioinformatics prediction website Targetscan was used to predict the relationship between miR-143-5p and eEF2, and dual luciferase reporter gene assay was carried out to confirm the prediction. As shown in Fig. 5a, miR-143-5p could target eEF2. In order to prove whether the eEF2 is a direct target gene of miR-143-5p, the recombinant plasmids of luciferase reporter vector-eEF2-Wt and eEF2-mut were first constructed, with inserted eEF2 mRNA3′-UTR. eEF2-mut referred to the mutation at the predicted binding site of eEF2 and miR-143-5p, which was used to identify the binding of miR-143-5p and eEF2. From a DLR gene system, in the miR-143-5p transfection group, the eEF2-3′-UTR co-transfection luciferase activity dropped by 42% approximately when compared to that of the other groups (*p* < 0.05) (Fig. 5b), while mutant eEF2-mut-3′-UTR luciferase activity showed no significant decrease (*p* > 0.05), which indicated that eEF2 was a potential target gene of miR-143-5p. Therefore, miR-143-5p inhibited the activity of eEF2.

### miR-143-5p-mediated eEF2 inhibition activates AMPK signaling pathway in NP cells

**Fig. 6:**
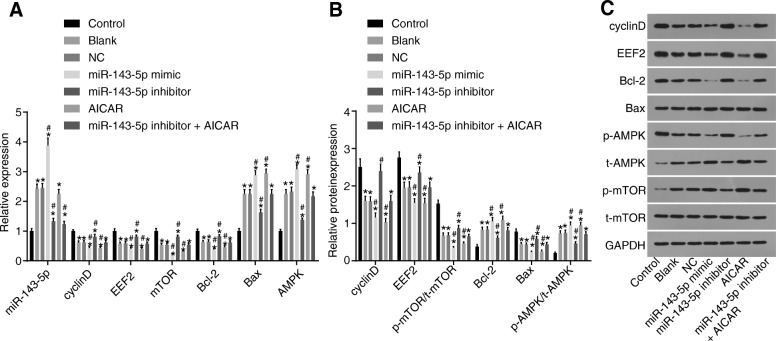
MiR-143-5p suppresses eEF2 and activates the AMPK signaling pathway in NP cells. **a** miR-143-5p expression and mRNA expression of cyclinD, eEF2, mTOR, Bcl-2, Bax, and AMPK in NP cells from degenerative IVD treated with miR-143-5p mimic, inhibitor, or AICAR determined by RT-qPCR. **b**, **c** Protein levels and bands of cyclinD, eEF2, mTOR, Bcl-2, Bax, and AMPK in NP cells treated with miR-143-5p mimic, inhibitor, or AICAR determined by Western blot analysis; AICAR was the activator of the AMPK signaling pathway; the experiment was repeated three times independently; data were expressed by mean ± standard deviation; comparison of data among multiple groups was analyzed using one-way ANOVA; ^*^, *p* < 0.05 vs. the control group (NP cells from normal IVD); ^#^, *p* < 0.05 vs. the blank group (NP cells from degenerative IVD without transfection) and the NC group (NP cells from degenerative IVD transfected with NC sequence); miR-143-5p mimic group refers to NP cells from degenerative IVD transfected with miR-143-5p mimic; miR-143-5p inhibitor group refers to NP cells from degenerative IVD transfected with miR-143-5p inhibitor; AICAR group refers to NP cells from degenerative IVD treated with AMPK signaling pathway activator, AICAR; miR-143-5p inhibitor + AICAR group refers to NP cells of degenerative IVD treated with AICAR and transfected with miR-143-5p inhibitor. NC, negative control; miR-143-5p, microRNA-143-5p; eEF2, eukaryotic translation elongation factor 2; AMPK, AMP activated protein kinase; Bax, Bcl-2 Associated X protein; Bcl-2, B cell lymphoma-2; mTOR, mammalian target of rapamycin; GAPDH, glyceraldehyde-3-phosphate dehydrogenase; NP, nucleus pulposus; ANOVA, analysis of variance

The potential effects of miR-143-5p targeting eEF2 on the APMK signaling pathway were analyzed using gain- and loss-of-function approaches. The NP cells from degenerative IVD were transfected with miR-143-5p mimic or inhibitor. RT-qPCR (Fig. 6a) showed the miR-143-5p expression was elevated after the NP cells from degenerative IVD transfected with miR-143-5p mimic while that was reduced after transfection with miR-143-5p inhibitor (*p* < 0.05). As shown by the obtained RT-qPCR and Western blot analysis results (Fig. 6a–c), when compared to those of the blank and NC groups, the mRNA and protein levels of Bax (apoptotic factor), and p-AMPK/t-AMPK protein level ratio were significantly increased, while the mRNA and protein levels of Bcl-2 (anti-apoptotic factor), cyclinD and eEF2, and p-mTOR/t-mTOR protein level ratio significantly decreased in the miR-143-5p mimic group (*p* < 0.05). However, in the miR-143-5p inhibitor group, the mRNA and protein levels of Bax and p-AMPK/t-AMPK protein level ratio were significantly decreased and that of Bcl-2, cyclinD and eEF2, and p-mTOR/t-mTOR protein level ratio were significantly elevated when compared to that of the blank and NC groups (*p* < 0.05). This data revealed that miR-143-5p negatively regulated eEF2 and activated the AMPK signaling pathway in NP cells. Additionally, AICAR was applied to induce the activation of AMPK signaling pathway. The mRNA and protein levels of those genes or proteins in the AICAR group exhibited similar results of the aforementioned genes or proteins as that in the miR-143-5p mimic group. In the miR-143-5p inhibitor + AICAR group, the expression of the abovementioned genes showed no significant difference when compared to that of the blank and NC groups (*p* > 0.05), suggesting that the contributory effect of miR-143-5p on NP cell apoptosis depended on the activation of the AMPK signaling pathway.

### Inhibition of miR-143-5p promotes NP cell differentiation by inactivating the AMPK signaling pathway

**Fig. 7:**
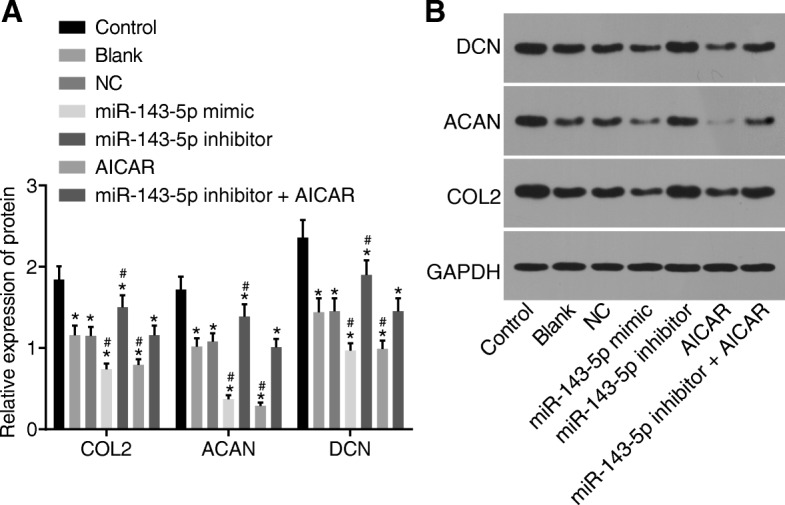
Inhibition of miR-143-5p facilitates NP cell differentiation by inactivating the AMPK signaling pathway. **a** Protein levels of CLO2, ACAN, and DCN in NP cells from degenerative IVD treated with miR-143-5p mimic, inhibitor, or AICAR. **b** Protein bands of CLO2, ACAN, and DCN in NP cells from degenerative IVD treated with miR-143-5p mimic, inhibitor, or AICAR; AICAR was the activator of the AMPK signaling pathway; the experiment was repeated three times independently; data were expressed by mean ± standard deviation; data among multiple groups were analyzed using one-way ANOVA; ^*^, *p* < 0.05 vs. the control group (NP cells from normal IVD); ^#^, *p* < 0.05 vs. the blank group (NP cells from degenerative IVD without transfection) and the NC group (NP cells from degenerative IVD transfected with NC sequence); miR-143-5p mimic group refers to NP cells from degenerative IVD transfected with miR-143-5p mimic; miR-143-5p inhibitor group refers to NP cells from degenerative IVD transfected with miR-143-5p inhibitor; AICAR group refers to NP cells from degenerative IVD treated with AMPK signaling pathway activator, AICAR; miR-143-5p inhibitor + AICAR group refers to NP cells of degenerative IVD treated with AICAR and transfected with miR-143-5p inhibitor. miR-143-5p, microRNA-143-5p; COL2, collagen type II; ACAN, aggrecan; DCN, decorin; GAPDN, glyceraldehyde-3-phosphate dehydrogenase; NC, negative control; AMPK, AMP activated protein kinase; NP, nucleus pulposus; ANOVA, analysis of variance

NP cell differentiation following transfection was assessed using Western blot analysis. As shown in Fig. 7, when compared to those of the control group, the protein levels of CLO2, ACAN, and DCN in NP cells were decreased in the remaining groups (*p* < 0.05). There was no obvious significance in protein levels of CLO2, ACAN, and DCN between the blank and NC groups (*p* > 0.05). When compared to those of the blank and NC groups, the protein levels of CLO2, ACAN, and DCN were significantly elevated in the miR-143-5p inhibitor group (*p* < 0.05) while significantly reduced in the miR-143-5p mimic and AICAR groups (*p* < 0.05). The protein levels of CLO2, ACAN, and DCN were significantly diminished in the miR-143-5p inhibitor + AICAR group as compared with those in the miR-143-5p inhibitor group (*p* < 0.05), while also displaying no clear difference when compared to those of the blank group (*p* > 0.05). The data suggested that inhibition of miR-143-5p promoted the differentiation of NP cells via inactivation of the AMPK signaling pathway.

### Inhibition of miR-143-5p induces NP cell proliferation via the inactivation of the AMPK signaling pathway

**Fig. 8:**
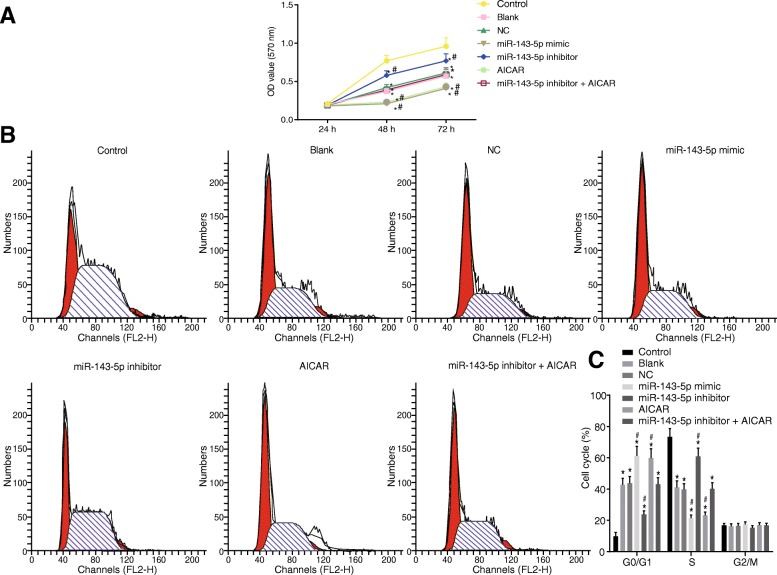
Inhibition of miR-143-5p increases NP cell proliferation and cell cycle entry by inactivating the AMPK signaling pathway. **a** The proliferation of NP cells from degenerative IVD treated with miR-143-5p mimic, inhibitor, or AICAR. **b**, **c** Cell cycles following treatment with miR-143-5p mimic, inhibitor or AICAR; AICAR was the activator of the AMPK signaling pathway; the experiment was repeated three times independently; data were expressed by mean ± standard deviation; data among multiple groups were analyzed using one-way ANOVA; *, *p* < 0.05 vs. the control group (NP cells from normal IVD); #, *p* < 0.05 vs. the blank and NC groups (NP cells from degenerative IVD without transfection and transfected with NC sequence, respectively); miR-143-5p mimic group refers to NP cells from degenerative IVD transfected with miR-143-5p mimic; miR-143-5p inhibitor group refers to NP cells from degenerative IVD transfected with miR-143-5p inhibitor; AICAR group refers to NP cells from degenerative IVD treated with AMPK signaling pathway activator, AICAR; miR-143-5p inhibitor + AICAR group refers to NP cells of degenerative IVD treated with AICAR and transfected with miR-143-5p inhibitor. NC, negative control; OD value, optical density value; miR-143-5p, microRNA-143-5p; AMPK, AMP activated protein kinase; NP, nucleus pulposus; ANOVA, analysis of variance

NP cell proliferation was measured by means of MTT assay, and the results (Fig. 8a) indicated that in contrast to the cell proliferation rate at the 24th hour, the cell proliferation rate at the 48th hour and 72nd hour displayed significant difference in each group (*p* < 0.05). When compared to that of the control group, the cell viability was significantly decreased in the miR-143-5p mimic, miR-143-5p inhibitor, AICAR, and miR-143-5p inhibitor + AICAR groups (*p* < 0.05). There was no significant difference in NP cell viability between the blank and NC groups (*p* > 0.05). When compared to that of the blank group and NC group, the cell viability decreased in the miR-143-5p mimic and AICAR groups (*p* < 0.05), and significantly increased in the miR-143-5p inhibitor group (*p* < 0.05). Besides, there was no difference in the miR-143-5p inhibitor + AICAR group (*p* > 0.05).

Flow cytometry analysis was further carried out to confirm the cell cycle, the results of which in Fig. 8 b, c suggested that, when compared to the control group, the blank, NC, miR-143-5p mimic, AICAR, miR-143-5p inhibitor, and miR-143-5p inhibitor + AICAR groups exhibited prolonged G0/G1 phase (increased cell proportion at G0/G1 phase), but shortened S phase (decreased cell proportion at G0/G1 phase) (*p* < 0.05). When compared to that of the blank and NC groups, G0/G1 phase shortened (cell proportion decreased) and S phase lengthened (cell proportion increased) in the miR-143-5p inhibitor group, while G0/G1 phase lengthened (cell proportion increased). At the same time, the S phase was shortened (cell proportion decreased) in the miR-143-5p mimic and AICAR groups (*p* < 0.05), and no significant difference was found in the miR-143-5p inhibitor + AICAR group (*p* > 0.05). There was no significant difference in cell proportion in the G2 phase among all groups (*p* > 0.05). Based on these results, it was concluded that miR-143-5p depletion promoted NP cell proliferation and cell cycle entry by inhibiting the AMPK signaling pathway.

### Inhibition of miR-143-5p suppresses NP cell apoptosis and senescence by inactivating the AMPK signaling pathway

**Fig. 9:**
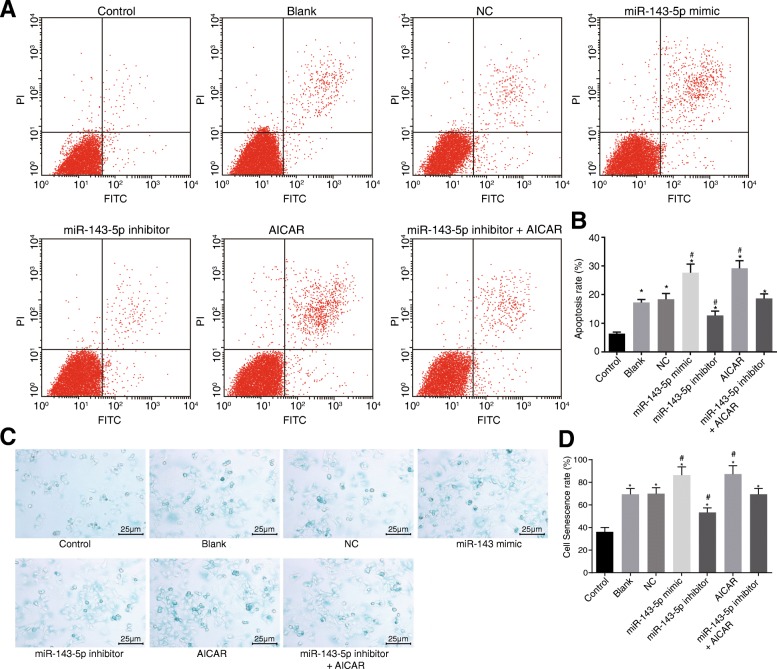
Inhibition of miR-143-5p suppresses NP cell apoptosis and senescence via inactivation of the AMPK signaling pathway. **a** The apoptosis of NP cells from degenerative IVD treated with miR-143-5p mimic, inhibitor or AICAR. **b** The percentage of apoptotic cells from degenerative IVD treated with miR-143-5p mimic, inhibitor or AICAR. **c** Senescent NP cells from degenerative IVD treated with miR-143-5p mimic, inhibitor or AICAR under an optical microscope (× 400). **d** SA-β-gal staining images of senescent NP cells from degenerative IVD treated with miR-143-5p mimic, inhibitor, or AICAR; the experiment was repeated three times independently; data were expressed by mean ± standard deviation; data among multiple groups were analyzed using one-way ANOVA; *, *p* < 0.05 vs. the control group (NP cells from normal IVD); #, *p* < 0.05 vs. blank and NC groups (NP cells from degenerative IVD without transfection and transfected with NC sequence, respectively); miR-143-5p mimic group refers to NP cells from degenerative IVD transfected with miR-143-5p mimic; miR-143-5p inhibitor group refers to NP cells from degenerative IVD transfected with miR-143-5p inhibitor; AICAR group refers to NP cells from degenerative IVD added with 0.5 mmol/L AICAR, AMPK signaling pathway activator; miR-143-5p inhibitor + AICAR group refers to NP cells of degenerative IVD added with 0.5 mmol/L AICAR and transfected with miR-143-5p inhibitor. NC, negative control; miR-143-5p, microRNA-143-5p; FITC, fluorescein isothiocyanate; NP, nucleus pulposus; SA-β-gal, senescence-associated β-galactosidase; ANOVA, analysis of variance

Flow cytometry analysis was also employed to test cell apoptosis. The results showed that when compared to that of the control group, the apoptotic rate was significantly increased in the blank, NC, miR-143-5p mimic, miR-143-5p inhibitor, AICAR, and miR-143-5p inhibitor + AICAR groups (*p* < 0.05). When compared to that of the blank and NC groups, cell apoptotic rate was reduced in the miR-143-5p inhibitor group and increased in the miR-143-5p mimic and AICAR groups (*p* < 0.05). There were no significant differences in cell apoptotic rate among the miR-143-5p inhibitor + AICAR, blank, and NC groups (*p* > 0.05) (Fig. 9a, b).

SA-β-gal staining was carried out to observe NP cell senescence. From the results of SA-β-gal staining, the cytoplasm of NP cells from normal IVD presented dark blue without any obvious vacuoles, but no obvious staining in nucleus was observed under the optical microscope (Fig. 9c, d). Positive rate in NP cells from degenerative IVD was significantly higher than that in the NP cells from normal IVD (*p* < 0.05). When compared to that of the control group, cell senescence significantly increased in the remaining groups *(p* < 0.05). In contrast to the blank and NC groups, cell senescence was diminished in the miR-143-5p inhibitor group *(p* < 0.05), while significantly increased in the miR-143-5p mimic and AICAR groups (*p* < 0.05). No significant difference was observed in the cell senescence among the blank, NC, and miR-143-5p inhibitor + AICAR groups (*p* > 0.05). Taken together, miR-143-5p inhibition impeded NP cell apoptosis and senescence by inhibiting the AMPK signaling pathway.

## Discussion

Human IDD is associated with dysfunction of NP cells, including differentiation, migration, proliferation, and apoptosis, and epigenetic processes and genetic background also are risk factors of IDD [28]. Furthermore, abnormal expression of miRNAs has been linked with various musculoskeletal disorders and also has a vital role to play in the pathogenesis of IDD [29]. However, the detailed mechanism of miR-143-5p in IDD remains poorly understood. The purpose of this study was to illustrate that miR-143-5p targeting the eEF2 gene could mediate IDD. Collectively, the data of this study revealed that miR-143-5p was highly expressed in rats with IDD while the inhibition of miR-143-5p was observed to inhibit NP cell senescence and apoptosis and promote cell proliferation and differentiation via repressing the activity of the AMPK signaling pathway.

Initially, based on the findings of this study, miR-143-5p was found to be expressed at a high level in degenerative IVD. Recent studies have confirmed that miR-143 is a muscle-enriched miRNA capable of mediating the myoblast differentiation and muscle aging [30]. Another previous study noted that the overexpression of miR-143-5p accelerates cell migration, proliferation, and melanogenesis in alpaca melanocyte in melanoma [31]. In addition, silencing miR-143-5p contributes to the differentiation dental pulp stem cells into odontoblasts by enhancing Runx2 expression via the OPG/RANKL signaling pathway [32]. Another important finding of this present study was that miR-143-5p suppressed NP cell proliferation and differentiation but promoted cell apoptosis and senescence in IDD. Consistently, a prior study also revealed upregulated miR-143 expression in degenerative disc tissues and restored miR-143 expression could promote IDD via negatively regulating Bcl-2 [16]. The results from the dual luciferase reporter gene assay confirmed that miR-143-5p targeted and negatively regulated eEF2. This study also found that eEF2 was expressed at a low level in degenerative IVD. eEF2, belonging to the GTP-binding translation elongation factor family, played a significant role in protein synthesis [33]. Recent evidence has indicated that the downregulation of eEF2 induced by IL-6 can cause impairment on myogenic differentiation during muscle homeostasis [34]. Furthermore, overexpression of eEF2 contributes to the inhibition of cardiomyocyte apoptosis during myocardial ischemia reperfusion via upregulating Bcl-2 expression [35].

In the following experiments, it was found that NP cells displayed a decreased expression ratio of p-mTOR/t-mTOR and an increased expression ratio of p-AMPK/t-AMPK following transfection with miR-143-5p mimic. The phosphorylation of the AMPK substrates was shown to be associated with upregulation of miR-143/145 in regulating the angiotensin-converting enzyme [36]. EEF2K is the enzyme that inactivates eEF2, which is activated by AMPK [20, 21]. It should be noted that mTOR is a protein kinase that participates in translation control and long-lasting synaptic plasticity [37], and it also played a significant role in cell senescence [38]. Furthermore, a previous study has suggested that AMPK signaling pathway is capable of inhibiting mTOR activity [39]. When the AMPK signaling pathway was activated by AICAR, NP cell proliferation and differentiation were inhibited but NP cell apoptosis and senescence were promoted. As a significant regulator of cell apoptosis [40], Bcl-2 has been confirmed to block apoptosis in degenerative IVD [16]. Bax is a member of Bcl-2 family [41]. Moreover, the stabilizing of the Bax/Bcl-2 ratio induces cell death and cell senescence [42]. It was also found that the cartilage differentiation-related genes COL2, ACAN, and DCN were suppressed when miR-143-5p was increased or the AMPK signaling pathway was activated. A recent study suggests that the COL2 protein level was increased when AMPK was poorly expressed [43]. It has also confirmed that DCN promotes nephron progenitor differentiation in the embryonic kidney [44]. The loss-of-function experiments further revealed that the inhibition of miR-143-5p blocked the AMPK signaling pathway and reversed the effects caused by the AMPK signaling pathway activation. Altogether, the inhibition of miR-143-5p promoted NP cell proliferation and differentiation yet restrained cell apoptosis and senescence by disrupting the AMPK signaling pathway.

## Conclusions

In conclusion, this study indicated that miR-143-5p was overexpressed in IDD. Furthermore, it has been shown that miR-143-5p promotes cell senescence and inhibits cell proliferation and differentiation in NP cells. Notably, eEF2 is a target gene of miR-143-5p, and miR-143-5p activates the AMPK signaling pathway in the regulation of IDD. It was speculated that miR-143-5p is a promising therapeutic target for IDD treatment. However, further studies are still required with more detailed methods to investigate more specific mechanism of miR-143-5p in IDD.

## Abbreviations

AMPK: AMP-kinase
ANOVA: Analysis of variance
BCA: Bicinchoninic acid
Bcl-2: B cell lymphoma-2
BSA: Bovine serum albumin
Ca/CaM: Calcium/calmodulin
CT: Cycle threshold
DAB: Diaminobenzidine
DHI: Disc height index
DLR: Dual luciferase reporter
eEF2: Eukaryotic elongation factor 2
eEF2K: EEF2 kinase
ELISA: Enzyme-linked immunosorbent assay
FBS: Fetal bovine serum
GAPDH: Glyceraldehyde-3-phosphate dehydrogenase
HE: Hematoxylin-eosin
HSFs: Human hypertrophic scar fibroblasts
IDD: Intervertebral disc degeneration
IVD: Intervertebral disc
LBP: Low back pain
miRNAs: MicroRNAs
MRI: Magnetic resonance imaging
mTOR: Mammalian target of rapamycin
NC: Negative control
NP: Nucleus pulposus
OD: Optical density
PBS: Phosphate buffered saline
PI: Propidium iodide
RDHC: Rate of disc height change
RT-qPCR: Reverse transcription quantitative polymerase chain reaction
SDS-PAGE: Sodium dodecyl sulfate-polyacrylamide gel electrophoresis
